# A ultrasonic nomogram of quantitative parameters for diagnosing breast cancer

**DOI:** 10.1038/s41598-023-39686-2

**Published:** 2023-07-31

**Authors:** Cong Wang, Ying Che

**Affiliations:** Ultrasound Department of the First Affiliated Hospital of Dalian Medical University, No.222 Zhongshan Road, Xigang District, Dalian City, Liaoning Province China

**Keywords:** Cancer, Cancer screening

## Abstract

This study aimed to develop a nomogram through the collection of quantitative ultrasound parameters to predict breast cancer. From March 2021 to September 2022, a total of 313 breast tumors were included with pathological results. Through collecting quantitative ultrasound parameters of breast tumors and multivariate regression analysis, a nomogram was developed. The diagnostic performances, calibration and clinical usefulness of the nomogram for predicting breast cancer were assessed. A total of 182 benign and 131 malignant breast tumors were included in this study. The nomogram indicated excellent predictive properties with an AUC of 0.934, sensitivity of 0.881, specificity of 0.848, PPV of 0.795 and NPV of 0.841. The calibration curve showed the predicted values are basically consistent with the actual observed values. The optimum cut-off for the nomogram was 0.310 for predicting cancer. The decision curve analysis results corroborated good clinical usefulness. The model including BI-RADS score, SWE and VI is potentially useful for predicting breast cancer.

## Introduction

Breast cancer is a malignant tumor that occurs from the ductal epithelium and terminal ductal epithelium of the breast^1^. In developed countries of Europe and the United States, the incidence rate of breast cancer ranks first among female malignant tumors. In China, breast cancer has always been the first cause of death among women^2^. Up to now, the diagnosis of breast cancer is mainly based on clinical examination combined with anatomical imaging information, such as X-ray photography, magnetic resonance imaging, ultrasound, breast physical examination, etc. Although there are many detection methods, biopsy is the gold standard to accurately judge the benign and malignant tumors. However, biopsy costs are high, has certain damage to patients, and is not suitable for a large number of early screening patients^3^.

In the imaging examination of breast, ultrasonography has become the first choice for clinicians and patients because of its advantages of simplicity, convenience, economy, effectiveness and safety^4^. The American College of Radiology has developed a Breast Imaging Reporting and Data System (BI-RADS) according to the different growth patterns of breast tumors and ultrasound manifestations^5^. BI-RADS not only grades the benign and malignant degree of breast tumors, but also standardizes the description language of ultrasonic signs, which makes us diagnosis of breast more standardized^6^. However, as the conventional ultrasound BI-RADS grading is a visual judgment, it is greatly affected by the technical experience of ultrasound doctors. The consistency between observers is poor, the false positive rate is high, and the imaging manifestations of breast tumors overlap. These limitations lead to a considerable number of benign lesions being over diagnosed as bi-rads:4 grade, increasing unnecessary biopsies^7,8^.

With the continuous application of science and technology in the medical system, more and more ultrasound methods are used. In order to improve the level of breast tumor diagnosis, the joint application of multiple technologies has become one of the current research hotspots^9,10^. Articles about multimodal ultrasound diagnosis of breast cancer are many, but the nomogram including quantitative parameters has not been conducted yet. Therefore, our study aimed to develop a nomogram through the collection of quantitative ultrasound parameters to predict breast cancer.

## Methods

### Ethical approval

This retrospective study was approved by the review board of the First Affiliated Hospital of Dalian Medical University. All methods were performed in accordance with the relevant clinical research ethics committee and with those of the Code of Ethics of the World Medical Association (Declaration of Helsinki). Informed consent was waived, which was approved by the Ethics Committee of the First Affiliated Hospital of Dalian Medical University because the present study is retrospective.

### Patients

From March 2021 to September 2022, consecutive women with breast lesion were collected according to the following inclusion criteria: (1) no radiotherapy or chemotherapy before examination; (2) BI-RADS category, SWE and VI could be accurately obtained; (3) tumors were confirmed by pathology; (4) ultrasound was performed within the previous one month of biopsy or resection. Finally, a total of 313 solid lesions from 313 women (mean age, 47.0 ± 12.7 years [range, 15–79 years]) were included in this study.

### Ultrasound examinations

Aplioi900 (Canon Medical Systems Corporation, Tokyo, Japan) equipped with 10–18 MHZ linear array transducer was used. The ultrasound examinations were performed by the same radiologist with over 10 years of experience. Through B-mode images, breast lesions were classified as BI-RADS category. When using superb microvascular imaging (SMI) technology to observe blood flow, gently place the probe on the body surface to avoid pressing. The size of the sampling frame should try to include the breast tissue within 1cm of the mass and its surrounding area. The region of interest was drawn manually along the margin of the lesion with the maximum Doppler signals, and then vascular index (VI) was automatically calculated. VI is the percentage ratio between the pixels for the Doppler signal and those for the total lesion. When conducting shear wave elastography (SWE) examination, place the probe gently at the breast lesion to determine the area of interest (the whole lesion and the surrounding area with high hardness should be included as far as possible), instruct the subject to hold his breath for 3–5 s, obtain a stable SWE image and freeze it, use Q-Box trace software to trace the tumor edge, and automatically obtain the mean elastic modulus value (E-mean). All data are measured 3 times and averaged. The radiologist was not blinded to the patients’ clinical characteristics. Pathological results were confirmed by US-guided biopsy or surgery.

### Statistical analysis

SPSS 23.0 (Chicago, IL) and R sofware (version 3.4.3) were used to perform the statistical analysis. Chi square test was applied to categorical variables. If the continuous numerical variable conforms to the normal distribution, the T-test should be applied; if not, the Ranksum test should be applied. The statistical significance was defined as less than 0.05 with two-sided test. A logistic regression model was built using stepwise method. We established nomogram prediction model with rms package. At the same time, the caret package is used for bootstrap method for internal verification and a calibration curve was drawn with 1000 bootstraps resample. The pROC package was used to plot the ROC curves and calculate the best cut-off point and area under curve (AUC). The “DecisionCurve” package was used to perform Decision-Curve analysis (DCA).

## Results

### Basic information

**Figure 1:**
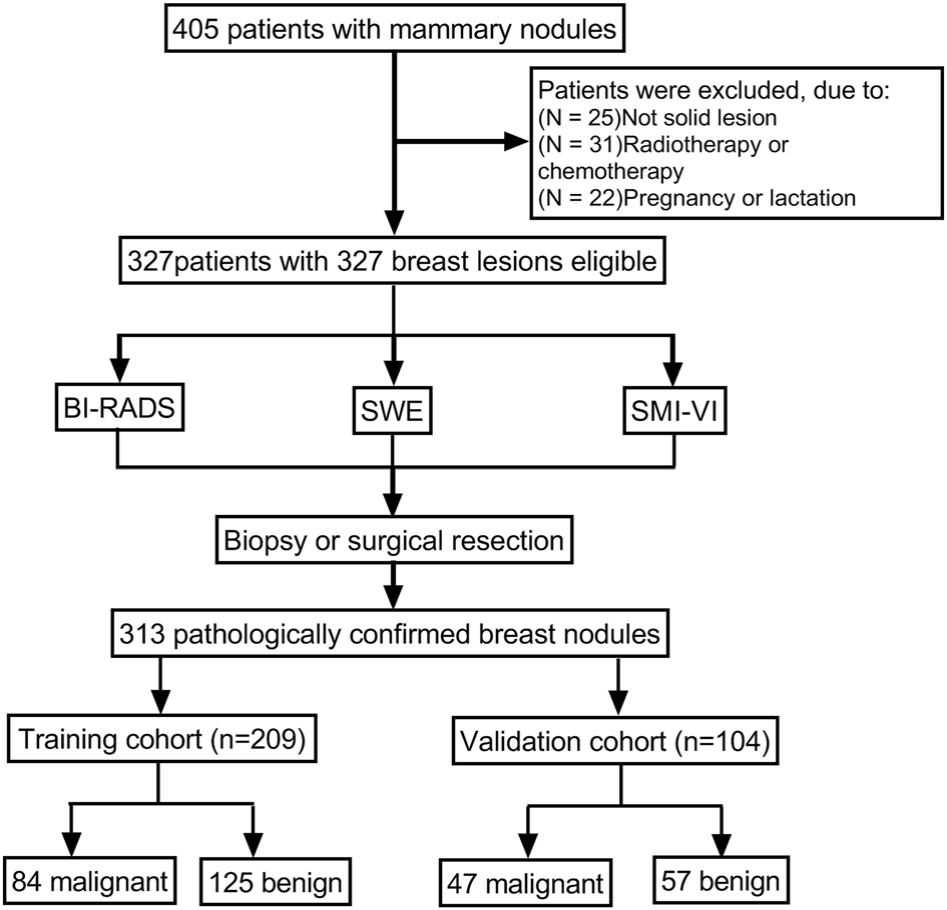
Flow chart of the study population enrolment.

**Figure 2:**
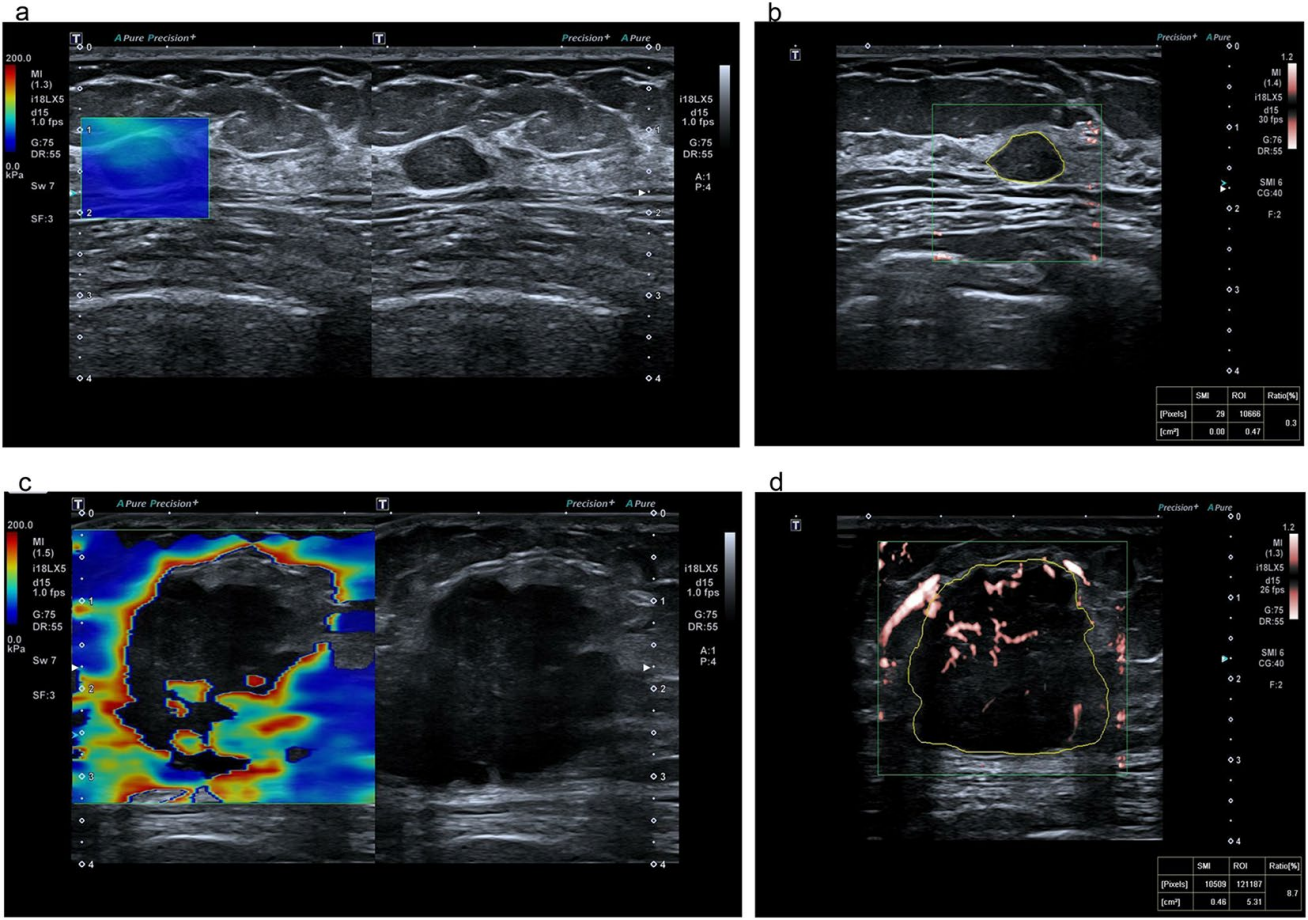
Examples of SWE and VI ultrasound images. A benign lesion with SWE, (**a**) 32.8 kPa and VI 0.3%, (**b**) and a malignant lesion with SWE, (**c**) 159.7 kPa and VI 8.7% (**d**).

125 benign and 84 malignant breast tumors in training group and 57 benign and 47 malignant breast tumors in validation group were analyzed and evaluated in this study (Fig. 1). The descriptions and univariate analysis of ultrasonic quantitative parameters are showed in Table 1. Symptom included palpable mass or nipple discharge. BI-RADS score, VI and Emean are significant factors for distinguishing benign and malignant breast tumors. Figure 2 demonstrates a benign and a malignant cases using ultrasonic quantitative parameters.

**Table 1:** Ultrasonic examination parameters in the training and validation groups.

	Training group				Validation group			
	Benign	Malignant	Test statistics	*P* value	Benign	Malignant	Test statistics	*P* value
Total	125	84			57	47		
BI-RADS (n/%)			Chisquare = 74.81	< 0.001			Chisquare = 35.01	< 0.001
3	51 (40.8)	5 (5.9)			23 (40.4)	3 (6.4)		
4a	54 (43.2)	20 (23.8)			25 (43.9)	12 (25.5)		
4b	13 (10.4)	18 (21.4)			5 (8.8)	9 (19.1)		
4c	7 (5.6)	27 (32.1)			4 (7)	14 (29.8)		
5	0 (0)	14 (16.7)			0 (0)	9 (19.1)		
VI (%)			Ranksum test	< 0.001			Ranksum test	< 0.001
Median (IQR)	3.3 (2.5,4.7)	5.1 (3.2,7.5)			3.1 (1.9,3.9)	4.7 (2.9,6.7)		
Emax (kPa)			Ranksum test	< 0.001			Ranksum test	< 0.001
Median (IQR)	31.7 (28,39.7)	65.8 (46.8,82.1)			35.5 (25,42.1)	59.4 (42.8,78.9)		
Age (year)			t-test = 7.82	< 0.001			t-test = 5.65	< 0.001
Mean (SD)	43.3 (11)	56.2 (12.6)			44.4 (10.5)	56.6 (11.4)		
Symptomatic (n/%)			Chisquare = 14.59	< 0.001			Chisquare = 5.03	< 0.05
No	54 (43.2)	15 (17.9)			21 (36.8)	8 (17)		
Yes	71 (56.8)	69 (82.1)			36 (63.2)	39 (83)

### Establishment and validation of nomogram model

**Figure 3:**
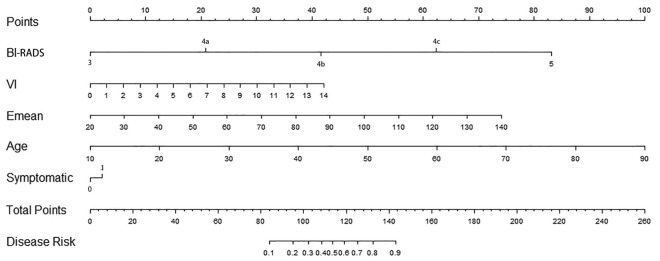
Nomogram including BI-RADS score, SWE and VI.

**Figure 4:**
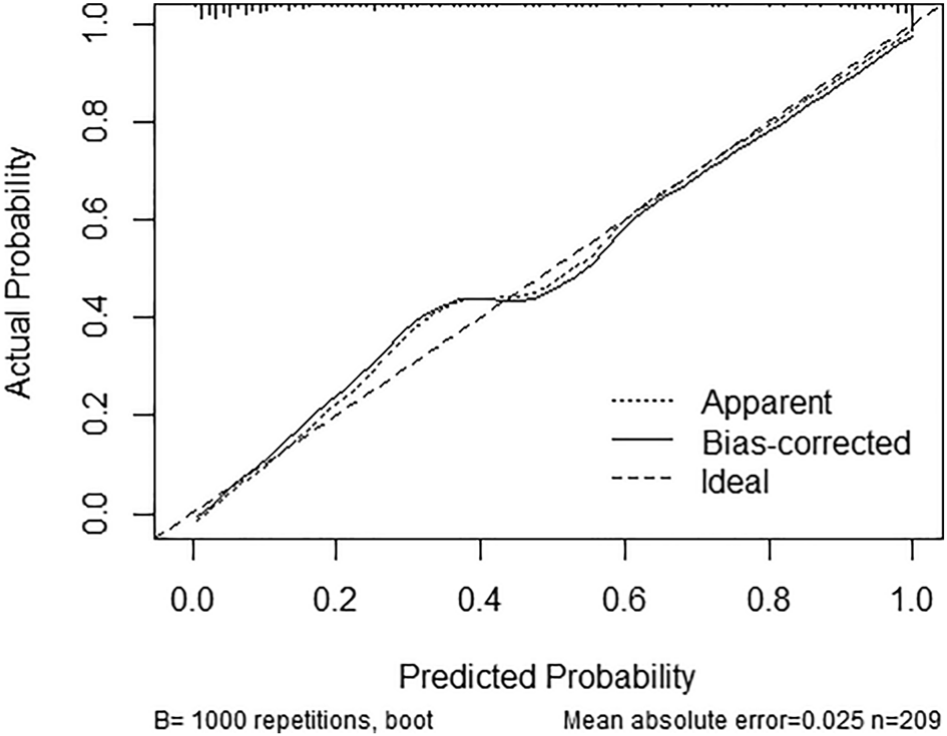
Calibration curves for the nomogram.

The nomogram model (Fig. 3) was established according to the results of binary multivariate logistic regression analysis. For example, a 45 year old woman had a palpable breast tumor with a BI-RADS grade of 4b, an Emean value of 90, and a VI of 7%. As shown in Fig. 1, a vertical line was drawn between the variable value and the corresponding point line, and the scores for age, symptom, BI-RADS, Emean, and VI were 42, 2, 41, 42, and 20, respectively. Thus, the total score was 147, and the risk of malignancy was greater than 0.9. Subsequently, the model was validated. The calibration curve was shown in Fig. 2. The predicted values are basically consistent with the actual observed values, which indicates that this nomogram model has good prediction ability (Fig. 4).

### Characteristics of prediction model

**Figure 5:**
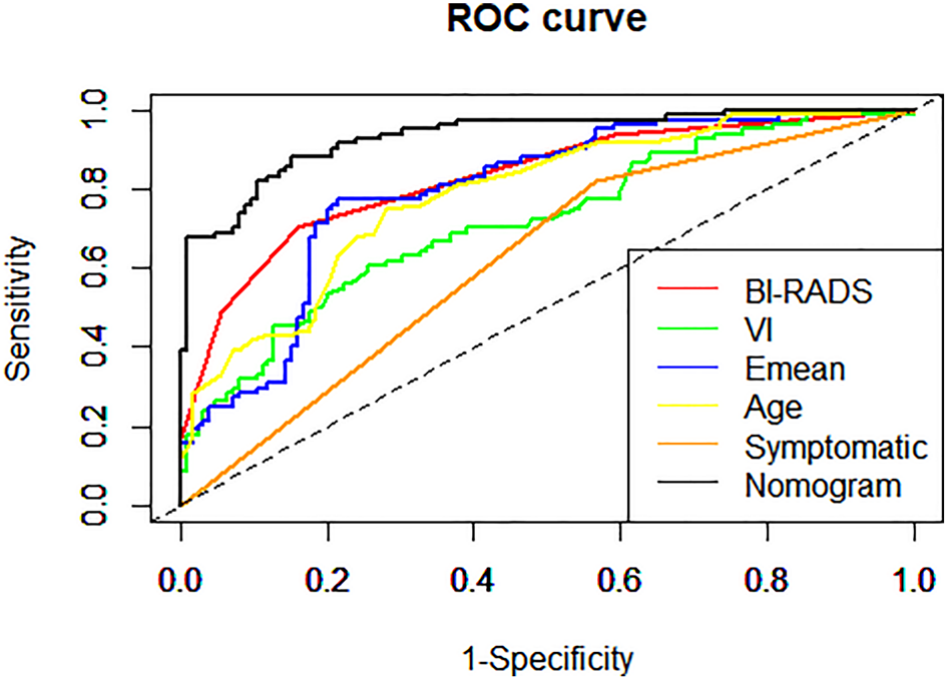
Receiver operating characteristic (ROC) curves of BI-RADS score, SWE, VI and the nomogram.

**Figure 6:**
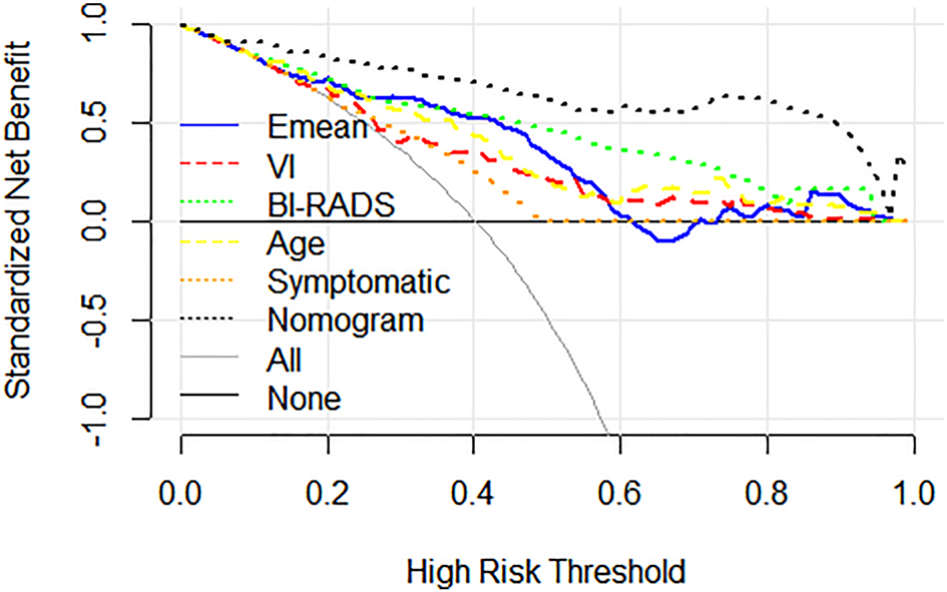
Decision curve analysis (DCA). The y-axis measures the net benefit. The gray line represents the assumption that all lesions are malignant. Te black line represents the assumption that all lesions are benign.

The ROC curve of the prediction model is shown in Fig. 5. The nomogram showed excellent predictive properties with an AUC of 0.934, sensitivity of 0.881, specificity of 0.848, PPV of 0.795 and NPV of 0.841 (Table 2). DCA was used to assess the clinical usefulness of the nomogram, which get higher benefit than BI-RADS category, VI and Emean (Fig. 6).

**Table 2:** Characteristics of prediction model based on Bootstrap internal validation.

	AUC	Cut-off	Sen	Spe	NPV	PPV
Nomogram	0.934	0.310	0.881	0.848	0.841	0.795
BI-RADS	0.831	> 4a	0.702	0.84	0.808	0.747
Emean	0.796	43.325	0.773	0.784	0.838	0.707
VI	0.717	4.61	0.607	0.744	0.738	0.614
Age	0.786	47.5	0.75	0.72	0.811	0.642
Symptomatic	0.627	0.5	0.821	0.432	0.771	0.493

PPV, positive predictive value; NPV, negative predictive value; PLR, positive likelihood ratio; NLR, negative likelihood ratio; AUC, area under the receiver operating characteristics.

## Discussion

In our study, a ultrasonic nomogram of quantitative parameters was developed to predict malignant and benign breast tumors. The model incorporating BI-RADS category, VI and Emean showed high sensitivity and specificity and owned good consistence between the actual observed values and prediction values for breast cancer. DCA showed our model has good clinical usefulness for predicting malignant and benign breast tumors.

Previous studies also have tried to develop nomogram to predict breast cancer on the basis of ultrasonic parameters. Luo et al. reported that the nomogram including the radiomics score and BI-RADS category was potentially useful for predicting breast malignancy^11^. Lang et al. developed a radiomics signature based on preoperative ultrasound to predict disease-free survival in patients with invasive breast cancer and assessed its additional value to the clinicopathological predictors for individualized disease-free survival prediction^12^. Meng et al. developed and validated a deep learning radiomic nomogram for assessing breast cancer pathological complete response after neoadjuvant chemotherapy based on the ultrasound images^13^. All of these new models were based on the computer-aided technology that converts ultrasound imaging information into a series of data through computer algorithms, and none of them attempted to develop a nomogram including the quantitative parameters of ultrasound itself. In the present study, we construct a model focusing on the collection of quantitative ultrasound parameters to predict breast cancer.

Our new nomogram model includes conventional ultrasound BI-RADS score, SWE and VI. SWE can quantitatively detect tissue hardness, and has the advantages of real-time, dynamic, non-invasive and good repeatability. The propagation speed of shear wave in different tissues is different. The faster the velocity is, the greater the hardness of biological tissues is. SWE has showed a good diagnostic performance and has many applications in the diagnosis of breast, thyroid, liver, kidney, prostate and blood vessel diseases^14,15^. As a new ultrasonic technology, SMI can quickly, conveniently and non-invasive detect the blood vessels in the tumor and observe the blood perfusion^16^. The introduction of the vascular index has enabled the quantitative evaluation of tumor vascularity in SMI^17^. The tumor blood supply determines the growth of the tumor. With the growth of the tumor, the blood vessels will extend more branches and form a large number of microvessels^18^. They will infiltrate and absorb a large amount of nutrients to the periphery for the survival of the tumor. At the same time, they will spread and metastasize far away. The VI of SMI is highly reproducible and improves diagnostic performance for differentiating between benign and malignant breast lesions^19,20^. However, the occurrence and development of all new technologies will be based on conventional ultrasound, which is very important in determining the malignant degree of breast lesions. In our study, the calibration curve of the nomogram showed high accuracy for individual predictions and the DCA showed that the nomogram added more benefit for predicting breast malignancy than either the treat-all scheme or the treat-none scheme.

There were several limitations in our current study. Firstly, bias can not be avoided as a retrospective study. Secondly, the data are still selective and cheap. Thirdly, we only carried out internal validation for this model. So It would be beneficial to test the nomogram multicenter, on a larger number of patients and prospectively.

## Conclusions

In our study, we constructed a new model including BI-RADS score, SWE and VI to diagnose breast cancer. The model could discriminate breast tumors between malignant and benign well.

## Data Availability

The datasets used and analyzed during the current study are available from the corresponding author on reasonable request.

## Abbreviations

AUC: Area under curve
BI-RADS: Breast imaging reporting and data system
DCA: Decision-curve analysis
E-mean: Mean elastic modulus value
SMI: Superb microvascular imaging
VI: Vascular index
SWE: Shear wave elastography

## References

[1] Sung H, Guo C, Li E, Li J, Pfeiffer RM, Guida JL (2019). The relationship between terminal duct lobular unit features and mammographic density among Chinese breast cancer patients. Int. J. Cancer..

[2] Ainvand MH, Shakibaei N, Ravankhah Z, Yadegarfar G (2021). Breast cancer incidence trends in Isfahan Province compared with those in England over the period 2001–2013. Int. J. Prev. Med..

[3] Zubor P, Kubatka P, Kajo K, Dankova Z, Polacek H, Bielik T (2019). Why the gold standard approach by mammography demands extension by multiomics? Application of liquid biopsy miRNA profiles to breast cancer disease management. Int. J. Mol. Sci..

[4] Hara Y, Yano H, Yamaguchi R, Iwasaki K (2021). Surgical excision of a lactating adenoma with rapid enlargement: A case report. Int. J. Surg. Case Rep..

[5] Mercado CL (2014). BI-RADS update. Radiol. Clin. North Am..

[6] Davis J, Liang J, Roh A, Kittrell L, Petterson M, Winton L (2021). Use of breast imaging-reporting and data system (BI-RADS) ultrasound classification in pediatric and adolescent patients overestimates likelihood of malignancy. J. Pediatr. Surg..

[7] Mohapatra SK, Mishra A, Sahoo TK, Nayak RB, Das PK, Nayak B (2021). The positive predictive values of the breast imaging reporting and data system (BI-RADS) 4 lesions and its mammographic morphological features. Indian J. Surg. Oncol..

[8] Assadi M, Velez E, Najafi MH, Gholamrezanezhad A (2019). The need for standardization of nuclear cardiology reporting and data system (NCAD-RADS): Learning from coronary artery disease (CAD), breast imaging (BI), liver imaging (LI), and prostate imaging (PI) RADS. J. Nucl. Cardiol..

[9] Zhou BY, Wang LF, Yin HH, Wu TF, Ren TT, Peng C (2021). Decoding the molecular subtypes of breast cancer seen on multimodal ultrasound images using an assembled convolutional neural network model: A prospective and multicentre study. EBioMedicine.

[10] Lee EJ, Chang YW (2020). Combination of quantitative parameters of shear wave elastography and superb microvascular imaging to evaluate breast masses. Korean J. Radiol..

[11] Luo WQ, Huang QX, Huang XW, Hu HT, Zeng FQ, Wang W (2019). Predicting breast cancer in breast imaging reporting and data system (BI-RADS) ultrasound category 4 or 5 lesions: A nomogram combining radiomics and BI-RADS. Sci. Rep..

[12] Xiong L, Chen H, Tang X, Chen B, Jiang X, Liu L (2021). Ultrasound-based radiomics analysis for predicting disease-free survival of invasive breast cancer. Front. Oncol..

[13] Jiang M, Li CL, Luo XM, Chuan ZR, Lv WZ, Li X (2021). Ultrasound-based deep learning radiomics in the assessment of pathological complete response to neoadjuvant chemotherapy in locally advanced breast cancer. Eur. J. Cancer..

[14] Ko KH, Jung HK, Park AY, Koh JE, Jang H, Kim Y (2020). Accuracy of tumor size measurement on shear wave elastography (SWE): Correlation with histopathologic factors of invasive breast cancer. Medicine (Baltimore).

[15] Pu H, Zhang XL, Xiang LH, Zhang JL, Xu G, Liu H (2019). The efficacy of added shear wave elastography (SWE) in breast screening for women with inconsistent mammography and conventional ultrasounds (US). Clin. Hemorheol. Microcirc..

[16] Zhong L, Wang C (2020). Diagnostic accuracy of ultrasound superb microvascular imaging for breast tumor: A meta-analysis. Med. Ultrason..

[17] Chae EY, Yoon GY, Cha JH, Shin HJ, Choi WJ, Kim HH (2021). Added value of the vascular index on superb microvascular imaging for the evaluation of breast masses: Comparison with grayscale ultrasound. J. Ultrasound. Med..

[18] Lee EJ, Chang YW, Oh E, Hwang J, Kim HJ, Hong SS (2021). Reproducibility and diagnostic performance of the vascular index of superb microvascular imaging in real-time breast ultrasonography for evaluating breast masses. Ultrasonography.

[19] Cai SM, Wang HY, Zhang XY, Zhang L, Zhu QL, Li JC (2020). The vascular index of superb microvascular imaging can improve the diagnostic accuracy for breast imaging reporting and data system category 4 breast lesions. Cancer Manag. Res..

[20] Uysal E, Öztürk M, Kilinçer A, Koplay M (2021). Comparison of the effectiveness of shear wave elastography and superb microvascular imaging in the evaluation of breast masses. Ultrasound. Q..

